# 18F-Flortaucipir (AV1451) imaging identifies grey matter atrophy in retired athletes

**DOI:** 10.1007/s00415-024-12573-0

**Published:** 2024-07-22

**Authors:** Anna Vasilevskaya, Chloe Anastassiadis, Simrika Thapa, Foad Taghdiri, Mozhgan Khodadadi, Namita Multani, Pablo Rusjan, Miracle Ozzoude, Apameh Tarazi, Asma Mushtaque, Richard Wennberg, Sylvain Houle, Robin Green, Brenda Colella, Neil Vasdev, Kaj Blennow, Henrik Zetterberg, Thomas Karikari, Christine Sato, Danielle Moreno, Ekaterina Rogaeva, David Mikulis, Karen Deborah Davis, Charles Tator, Maria Carmela Tartaglia

**Affiliations:** 1 Tanz Centre for Research in Neurodegenerative Diseases, University of Toronto, Krembil Discovery Tower, 60 Leonard Avenue, 6th Floor 6KD-407, Toronto, ON M5T 2S8 Canada; 2 Institute of Medical Science, University of Toronto, Toronto, ON Canada; 3 Division of Neurology, Toronto Western Hospital, University Health Network, Toronto, ON Canada; 4 Canadian Concussion Centre, Toronto Western Hospital, Krembil Brain Institute, University Health Network, Toronto, ON Canada; 5 Douglas Mental Health University Institute, Montreal, QC Canada; 6 Department of Psychiatry, McGill University, Montreal, QC Canada; 7 Brain Health Imaging Centre, Campbell Research Institute, Centre for Addiction and Mental Health, and Department of Psychiatry, University of Toronto, Toronto, ON Canada; 8 KITE Research Institute, University Health Network, Toronto, ON Canada; 9 Department of Psychiatry and Neurochemistry, Institute of Neuroscience and Physiology, The Sahlgrenska Academy at the University of Gothenburg, Mölndal, Sweden; 10 Clinical Neurochemistry Laboratory, Sahlgrenska University Hospital, Mölndal, Sweden; 11 Department of Neurodegenerative Disease, UCL Institute of Neurology, Queen Square, London, UK; 12 UK Dementia Research Institute at UCL, London, UK; 13 Hong Kong Center for Neurodegenerative Diseases, Clear Water Bay, Hong Kong, China; 14 Wisconsin Alzheimer’s Disease Research Center, University of Wisconsin School of Medicine and Public Health, University of Wisconsin-Madison, Madison, WI USA; 15 Division of Neuroradiology, Joint Department of Medical Imaging, University Health Network, Toronto, ON Canada; 16 Department of Surgery, University of Toronto, Toronto, ON Canada; 17 Division of Neurosurgery, Toronto Western Hospital, Krembil Brain Institute, University Health Network, Toronto, ON Canada

**Keywords:** Concussion, Athletes, Tau, Pet, Chronic traumatic encephalopathy, Neurodegeneration

## Abstract

**Background:**

The long-term consequences of concussions may include pathological neurodegeneration as seen in Alzheimer’s disease (AD) and chronic traumatic encephalopathy (CTE). Tau-PET showed promise as a method to detect tau pathology of CTE, but more studies are needed

**Objective:**

This study aimed (1) to assess the association of imaging evidence of tau pathology with brain volumes in retired athletes and (2) to examine the relationship between tau-PET and neuropsychological functioning.

**Methods:**

Former contact sport athletes were recruited through the Canadian Football League Alumni Association or the Canadian Concussion Centre clinic. Athletes completed MRI, [^18^F]flortaucipir tau-PET, and a neuropsychological battery. Memory composite was created by averaging the Rey Auditory Verbal Learning Test and Rey Visual Design Learning Test z-scores. Grey matter (GM) volumes were age/intracranial volume corrected using normal control MRIs. Tau-PET % positivity in GM was calculated as the number of positive voxels (≥ 1.3 standardized uptake value ratio (SUVR)/total voxels).

**Results:**

47 retired contact sport athletes negative for AD (age:51 ± 14; concussions/athlete:15 ± 2) and 54 normal controls (age:50 ± 13) were included. Tau-PET positive voxels had significantly lower GM volumes, compared to tau-PET negative voxels (− 0.37 ± 0.41 vs. − 0.31 ± 0.37, paired *p* = .006). There was a significant relationship between GM tau-PET % positivity and memory composite score (r =  − .366, *p* = .02), controlled for age, PET scanner, and PET scan duration. There was no relationship between tau-PET measures and concussion number, or years of sport played.

**Conclusion:**

A higher tau-PET signal was associated with reduced GM volumes and lower memory scores. Tau-PET may be useful for identifying those at risk for neurodegeneration.

**Supplementary Information:**

The online version contains supplementary material available at 10.1007/s00415-024-12573-0.

## Introduction

Most traumatic brain injuries (TBIs) are concussions and mild TBIs. Concussions and mild TBIs have been associated with neurodegeneration including Alzheimer’s disease (AD), chronic traumatic encephalopathy (CTE), amyotrophic lateral sclerosis, Parkinson’s disease, and dementia with Lewy bodies [1]. Both AD and CTE are tauopathies characterized by abnormal accumulation of hyperphosphorylated tau inclusions of mixed 3R/4R isoforms. Despite the similarities, the structure of tau implicated in CTE is distinct from that in AD[2]. [^18^F]Flortaucipir (a.k.a. Tauvid™, [^18^F]AV-1451 or [^18^F]T807) tau-PET is widely used in AD due to its high specificity to paired helical filament tau pathology in AD, but relatively low affinity in other tauopathies like frontotemporal lobar degeneration, progressive supranuclear palsy, and corticobasal degeneration [3].

There is currently no antemortem biomarker for the pathology of CTE and the utility of flortaucipir tau-PET as a biomarker for the tau pathology of CTE is still unclear. Early case reports examining flortaucipir tau-PET in former athletes did not show a consistent tau-PET signal pattern [4, 5]. A more recent larger study found higher flortaucipir tau-PET signal in bilateral superior frontal, bilateral medial temporal, and left parietal areas in 26 former athletes negative for AD pathology compared to 31 controls. Furthermore, a higher tau-PET signal was significantly associated with longer years of play [6]. The literature comparing the pattern of flortaucipir ligand binding to tau pathology of CTE is limited. One pathology series of 5 CTE cases of various severity reported that despite abundant tau aggregates in multiple regions of all CTE brains, only faint or no flortaucipir binding signal could be detected by autoradiography [7]. Another brief report on one amyloid-negative former athlete found a modest correlation between tau pathology of CTE and flortaucipir tau-PET completed 52 months prior to death [8]. A more recent study comparing tau immunohistochemistry to flortaucipir tau-PET autoradiography in brain tissue slices from 12 CTE cases ranging from stage I to IV found variable specific binding (68.7% ± 10.5%) of the tracer to CTE pathology [9]. Another recent study comparing a near end-of-life flortaucipir tau-PET with neuropathology in 4 CTE cases found a strong association between tau-PET signal and tau density in cortical and limbic areas [3].

In AD, flortaucipir is associated with atrophy and neuropsychological functioning [10]. Additionally, gross pathological examinations of brains with confirmed CTE were noted to have generalized cerebral atrophy predominantly in the frontal and temporal lobes, thalamus, hypothalamus, and mammillary body atrophy, enlarged lateral and third ventricles, and thinning of the corpus callosum and cavum septum pellucidum [11]. Similarly, premortem MRI findings of neuropathologically confirmed CTE cases found greater atrophy in orbitofrontal, dorsolateral frontal, superior frontal, anterior temporal, and medial temporal lobes, with larger third and lateral ventricles in CTE cases compared to MRI scans of healthy controls. Greater degree of tau neuropathology also corresponded to greater atrophy on MRI [12]. The relationship between flortaucipir binding and atrophy in former contact sports athletes without AD is unknown.

Therefore, the main objective of this study was to evaluate the effect of flortaucipir tau-PET ligand binding in a cohort of retired contact sport athletes negative for AD biomarkers. To this end, we investigated (1) the relationship between flortaucipir tau-PET signal and grey matter volumes; (2) the visual pattern of tau-PET signal in the grey matter; and (3) the relationship between the tau-PET signal and neuropsychological scores.

## Methods

### Participants

This study was approved by the Research Ethics Board of the University Health Network, and informed written consent was obtained from all participants. Former contact sport athletes were recruited through the Canadian Football League (CFL) Alumni Association or the Canadian Concussion Centre concussion clinic. The inclusion criteria for participants were that they were retired contact sports athletes under 85 years old and fluent in English. Exclusion criteria at the time of study visit were the diagnosis of a neurological or psychiatric disorder (i.e., epilepsy, stroke, major depression disorder, bipolar, schizophrenia), levels of phosphorylated tau at threonine 181 (p-tau181) in plasma and/or levels of amyloid-β 42, total tau, and p-tau in cerebrospinal fluid (CSF) consistent with those found in AD, systemic illnesses affecting the brain, or pathology other than white matter hyperintensities (WMH) seen on brain MRI scans. We used self-report questionnaires where participants were asked to recall their concussion history including concussion number and years of play (total and professional). Healthy controls were recruited from the community through advertising. Their data were only used for the creation of w-scores for the retired athletes. Exclusion criteria for the healthy controls were as follows: previous history of concussions, repetitive head impacts, or significant sport history, neurological or psychiatric disorder, other systemic illnesses affecting the brain, any significant lesions seen on brain MRI scans, or impaired performance on neuropsychological testing (i.e., normed scores > 1.5 SD below the mean).

### Neuropsychological assessments

All retired contact sport athletes completed a full clinical neuropsychological assessment battery. Neuropsych by domain assessment was completed that includes symptoms collected from cognitive, behavioral, and neuropsychiatric domains. Memory assessments included the Rey Auditory Verbal Learning Test (RAVLT) and Rey Visual Design Learning Test (RVDLT). Attention and speed of processing measures included Trail Making Test (TMT) part A, Digit span forward, Stroop Color Naming and Word Reading Tests, and the Symbol Digit Modalities Test (SDMT), both oral and written versions. Executive function assessments included the Digit span backwards, Wisconsin Card Sorting Test (WCST), and TMT part B[13]. The scores were standardized using established norms[14]. Higher normed scores on all cognitive measures represent better performance.

A series of composite scores were created for memory (RAVLT trials 1–5 z-score, RAVLT long-delay recall z-score, RVDLT trials 1–5 z-score), attention with simple and complex speed of processing (TMT part A z-score, Digit span forward z-score, Stroop Color Naming and Stroop Word Reading tests z-scores, and SDMT written and oral z-scores), and executive function (Digit span backwards t-score, WCST percent error t-score, WCST percent perseverative error t-score, WCST percent non-perseverative error t-score, WCST conceptual level responses t-score, and TMT-B t-score). Composite scores for each neuropsychological domain were created by adding similar scores of respective neuropsychological tests for each domain of interest, then averaging them out. For executive function domain specifically, the scores from the WCST were first averaged out before adding them to TMT-B t-score and creating a composite.

### Blood analysis

Plasma from the venous blood samples of retired contact sport athletes was analyzed for p-tau181 as a marker of AD pathology using one of the two of the following assays, as previous studies suggest it is specificity to AD and not CTE or athletes at risk for CTE including data from our own cohort [14–16]:

1. Using an in-house assay on an HD-1 Single molecule array (Simoa) instrument (Quanterix, Billerica, MA), as previously described [16]. Samples were diluted two-fold with assay diluent and analyzed as singlicates. Quality control samples (QCs) were analyzed in duplicates at the start and the end of each plate and used to assess analytical reproducibility. The intra-run and inter-run coefficients of variation (CV) were both < 20%. The threshold for AD positivity in plasma p-tau181 levels among the retired athletes was based on published levels in cognitively unimpaired healthy adults [16, 17]. For young adults (< 60 y.o.) the threshold of > 10.5 pg/mL of plasma p-tau181 was considered AD biomarker positive. For older adults (≥ 60 y.o.), > 13.3 pg/mL of plasma p-tau181 was considered AD biomarker positive [16, 17].
2. Using commercially available Simoa p-tau181 Advantage V2 Kit (an assay based on the previously described method) [16] on an SR-X Simoa instrument. Plasma samples were analyzed following the manufacturer’s instructions. In brief, samples were diluted four-fold with assay diluent and analyzed as duplicates. QCs were analyzed in duplicates, and the intra-run and inter-run reproducibility were both < 20%. The threshold for AD positivity in p-tau181 levels was set at > 2.2 pg/mL [18].

### Cerebrospinal fluid (CSF) analysis

Lumbar punctures for cerebrospinal fluid (CSF) collection were completed following the Alzheimer’s Disease Neuroimaging Initiative (ADNI) protocol [19]. CSF was placed into polypropylene tubes, and levels of amyloid-β 42 (Aβ42; Innotest β-amyloid (1–42), Fujirebio), p-tau (Innotest phospho-tau (181p), Fujirebio), and total tau (t-tau; Innotest hTAU-Ag, Fujirebio) were measured using sandwich ELISAs. AD pathology was deemed present if p-tau > 68 pg/mL and Aβ42 to t-tau index < 0.8 [20].

### Structural MRI acquisition

All structural scans were acquired using a 3 T MRI scanner (GE Signa DHx, Milwaukee, WI, USA) with an 8-channel head coil. The T1-weighted structural MRI scans were acquired using inversion recovery spoiled gradient echo (IR-SPGR) in the sagittal plane using the following parameters: TE = 2.8 ms, TR = 7 ms, flip angle = 11°; 176 slices, slice thickness = 1.2 mm, 256 × 256 matrix, and FOV = 26 cm.

### Voxel-based morphometry (VBM)

VBM analysis was performed using the CAT12 toolbox (http://www.neuro.uni-jena.de/cat/index.html) of the SPM12 software (https://www.fil.ion.ucl.ac.uk/spm/software/spm12/) running in Matlab R2018a (MathWorks, Natick, MA). For VBM, the structural imaging data were preprocessed using the default pipeline of CAT12 toolbox. Briefly, structural images were noise and bias corrected for MRI inhomogeneities, segmented into grey matter, white matter, and CSF, and spatially normalized. Finally, the preprocessed grey matter image volumes were smoothed with an 8 mm full width at half maximum (FWHM) isotropic Gaussian kernel.

### PET acquisition and PET processing

PET imaging with 5 mCi of [^18^F]Flortaucipir ([F-18]AV1451, [F-18]T807; AVID Radiopharmaceuticals) was completed at the CAMH Brain Health Imaging Centre as previously described [21]. Participants were scanned on either a Biograph HiRez XVI PET/CT scanner (Siemens Molecular Imaging, Knoxville, TN, USA) or on a High-Resolution Research Tomograph (HRRT) (CPS/Siemens, Knoxville, TN, USA) PET scanner. The detailed acquisition parameters and analysis pipeline are described elsewhere[22]. Following a 45-min uptake time, emission PET data were acquired in list mode for 75 min. Region of interest analysis of the PET data was completed using in-house ROMI software [23] and Statistical Parametric Mapping version 8 (SPM8; https://www.fil.ion.ucl.ac.uk/spm/software/spm8/). The PET images were corrected for head motion and partial volume effect [24]. Regions of interest included inferior cerebellar grey matter using a native ROMI atlas, and a whole cortical grey matter mask that was created using LONI Probabilistic Atlas (LPBA40; freely available from https://loni.usc.edu/research/atlases). Standardized uptake value ratios (SUVRs) were calculated for full grey matter (excluding cerebellum) using inferior cerebellar grey matter as a reference region and averaged for the 80–100 min (or 80–90 min in those participants who only had a shorter scan available for analysis) post-tracer injection time frame.

Because of differences in original spatial resolution between MRI and PET data, a different smoothing kernel was applied to PET data to equalize the spatial resolution [25]. Tau-PET images from the Biograph HiRez XVI PET/CT scanner were smoothed using 6.55 × 6.55 × 7.75 (x, y, z) mm FWHM Gaussian kernel, while tau-PET images from the HRRT scanner were smoothed using a 7.37 × 7.37 × 7.91 (x, y, z) mm FWHM Gaussian kernel. A tau-PET threshold of > 1.30 SUVR was used to designate positive voxels [26, 27]. Region-specific tau-PET % positivity was calculated as a ratio of the number of positive voxels to the total number of voxels.

### Grey matter w-score analysis

**Fig. 1:**
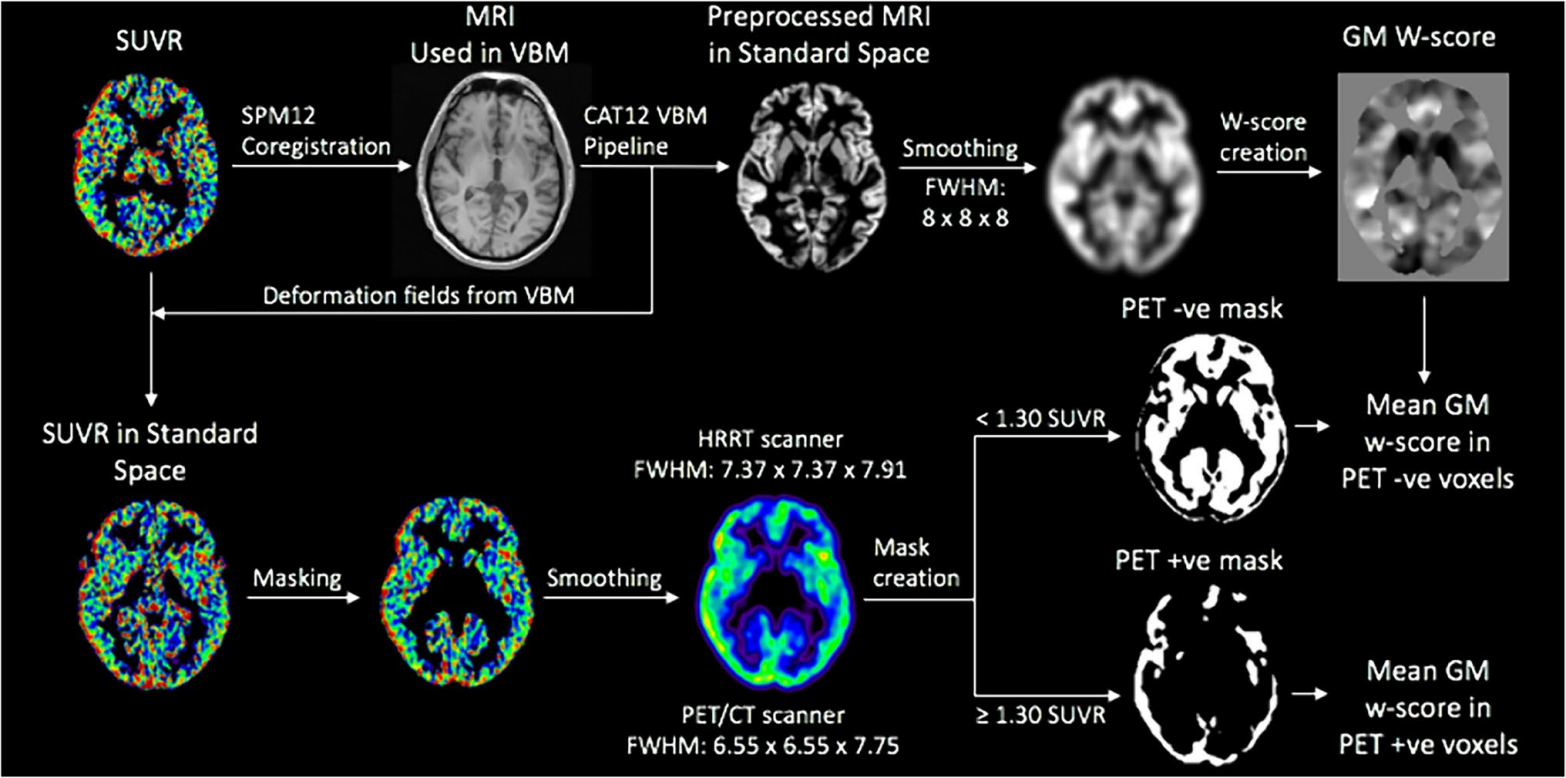
A neuroimaging approach to extract mean grey matter volumes from tau-PET positive and negative voxels of each participant. Illustration of the steps required to extract the mean standardized grey matter volumes (VBM w-scores) from tau-PET positive and negative voxels for each participant. First, motion and partial volume effect corrected SUVR images are created using inferior cerebellar grey matter as a reference region. The SUVR image is then co-registered to the MRI image that is preprocessed using the standard CAT12 VBM pipeline. Next, the deformation fields from MRI are used on the SUVR image to bring it into standard space, followed by masking using a grey matter mask created from LONI Probabilistic Atlas (LPBA40) and smoothing. Different smoothing kernels were applied depending on the PET scanner used: subjects scanned on the HRRT scanner had a smoothing kernel of FWHM: 7.37 × 7.37 × 7.91; subjects scanned on the PET/CT scanner had a smoothing kernel of FWHM: 6.55 × 6.55 × 7.75. Finally, the smoothed SUVR images were threshold at ≥ 1.30 to create a mask with tau-PET positive voxels, and < 1.30 to create a mask with tau-PET negative voxels. The participant-specific positive and negative tau-PET masks were overlaid over the VBM w-score images and mean GM w-score values were extracted for each participant. *SUVR* Standardized uptake value ratios, *VBM* voxel-based morphometry, *GM* grey matter

W-scores are z-scores that are adjusted for specific covariates [25]. A summary of the w-score value analysis is summarized in Fig. 1. To control for the effects of age and total intracranial volume (TIV) on grey matter volumes in retired athletes, w-score maps were created for each individual participant using MRI scans from the healthy control cohort using previously published methods [25]. The anatomical MRI scans from healthy controls and retired athletes used in the w-score creation were first processed using CAT12 VBM pipeline described previously in the methods section of this paper. Then, voxelwise regressions were performed between MRI images in healthy controls with age and TIV values using SPM12 software. W-score maps for each retired athlete were computed using the following formula: w-score = [(retired athlete’s MRI) − (retired athlete’s MRI predicted for their age and TIV)/SD of the residuals in healthy controls [25]. Average w-score values were then extracted from the tau-PET positive grey matter mask and tau-PET negative grey matter mask for each retired athlete before statistical analysis. Lower w-score values were reflective of lower grey matter volumes.

### Tau-PET quartile creation

To visualize patterns of tau-PET signal, % positivity values in the total grey matter were divided into 4 quartiles following a similar previously published approach, resulting in 4 groups [28]. The first quartile contained participants with the lowest % positivity values, while the fourth quartile contained the highest % positivity values. Tau-PET grey matter SUVR images of participants within each quartile were then averaged and overlaid over spm152 standard brain using MRIcroGL V13.2 (https://www.nitrc.org/projects/mricrogl).

### Statistical analyses

Paired *t*-tests were used to compare grey matter volume w-score values within subjects. Pearson partial correlations controlled for age, education, tau-PET scanner, and scan duration were used to investigate the associations between tau-PET measures and neuropsychological scores. To check which areas of the grey matter sequentially increased the greatest in tau-PET % positivity, multiple linear regressions were run with region-specific voxel extent as a dependent variable and quartile group and age as independent variables. The regions were then ordered based on unstandardized coefficients. To control for influence of outliers, linear regressions were performed with 95% bias-corrected accelerated (BCa) bootstrapped CIs with 2000 repetitions stratified by quartile group. All analyses were controlled for multiple comparisons using Bonferroni.

## Results

### Participants

A total of 106 participants were recruited for this study:

1. Fifty-two retired contact sports athletes had blood p-tau181 or CSF analysis for the presence of AD pathology. Every athlete participant was screened for the presence of AD pathology using either: (1) blood p-tau181; (2) CSF measures of Aβ42, p-tau, and t-tau; or (3) both blood p-tau181 and CSF markers of Aβ42, p-tau, and t-tau. Out of 52 athletes, five were excluded from this analysis: four were positive for AD based on plasma p-tau181 values of 13.18 pg/ml (Simoa pTau-181 V1 Kit), 7.4 pg/ml, 2.8 pg/ml, and 2.5 pg/ml (Simoa pTau-181 V2 Kit), and one athlete had tau-PET pattern of retention suggestive of AD and total grey matter SUVR of 2.73. The remaining 47 athletes [age (Min = 24, Max = 85, Mean = 51, S.D. = 14 years); sex (44 males, 3 females)] were negative for AD and the cohort descriptors are summarized in Table 1. A portion of the retired athletes cohort (10/47, 21.3%) only had a shorter scan time of 80–90 min post-tracer injection available for analysis. More than half of the retired athletes cohort (27/47 (57.4%)) had a family history of dementia.
2. Fifty-four healthy controls [age (Min = 26, Max = 71, Mean = 50, S.D. = 13 years); sex (41 males, 13 females)] were included to create VBM w-scores for the athletes cohort.

**Table 1:** Athletes cohort characteristics

	Cohort descriptors (N = 47)
Age, years	51 (14)
Sex	44 Males: 3 Females
Concussion #	Min = 0; Max = 35 Median = 5
Education, years	15 (2)
Sports played	Football: 33
	Hockey: 8
	Boxing: 4
	Soccer: 1
	Snowboarding: 1
Highest level of play	High School: 0
	College: 4
	Semi-professional: 1
	Professional: 42
Played professionally, years	7 (5)^a^
Total years played, years	17 (6)^b^
APOE4 carriers, N (%)	12 (25.5%)
Family history of dementia, N (%)	27 (57.4%)
*Tau-PET*	
Scanner	
PET/CT, N (%*)*	31 (66%)
HRRT, N (%)	16 (34%)
Scan time for SUVR creation	
80–100 min post-injection, N (%)	37 (78.7%)
80–90 min post-injection, N (%)	10 (21.3%)
*Plasma*	
Biofluid visit, N	2 years after tau-PET: 2
	Same visit as tau-PET: 22
	2 years prior to tau-PET: 5
	4 years prior to tau-PET: 2
	6 years prior to tau-PET: 1
P-tau181 (in-house assay), pg/mL	9.30 (2.10)
*N* (%)	14 (29.8%)
P-tau181 (commercial assay), pg/mL	1.11 (0.48)
*N* (%*)*	18 (38.3%)
*CSF*	
Biofluid visit, N	2 years after tau-PET: 1
	Same visit as tau-PET: 17
T-tau, pg/mL	255.87 (130.98)
P-tau181, pg/mL	37.12 (11.40)
Aβ42, pg/mL	926.43 (410.90)
*N* (%)	18 (38.3%)

Values are presented as mean (standard deviation) unless otherwise specified. SUVR, standardized uptake value ratio; t-tau, total tau; p-tau181, phosphorylated tau 181; Aβ42, amyloid beta 42

^a^Data available for 44 participants

^b^Data available for 36 participants

### Biofluid AD biomarker concordance

Eleven retired athletes had fluid biomarkers (CSF and/or blood) analyzed at more than one visit. The detailed data on biomarker analysis results are included in Supplementary Table 1. Ten of these 11 athletes were negative for AD pathology across all of their visits; with 1 retired athlete having plasma p-tau181 values consistent with the presence of AD pathology four years post-tau-PET visit, and therefore, this participant was excluded from this study.

### Tau-PET and sports history

There was no significant correlation between tau-PET total grey matter % positivity or tau-PET total grey matter SUVR and concussion number, number of years played professionally, total years played, or the total symptoms from the memory, executive function, or behavior domains, controlled for age, tau-PET scanner, and scan duration.

### Grey matter volumes and tau-PET

**Fig. 2:**
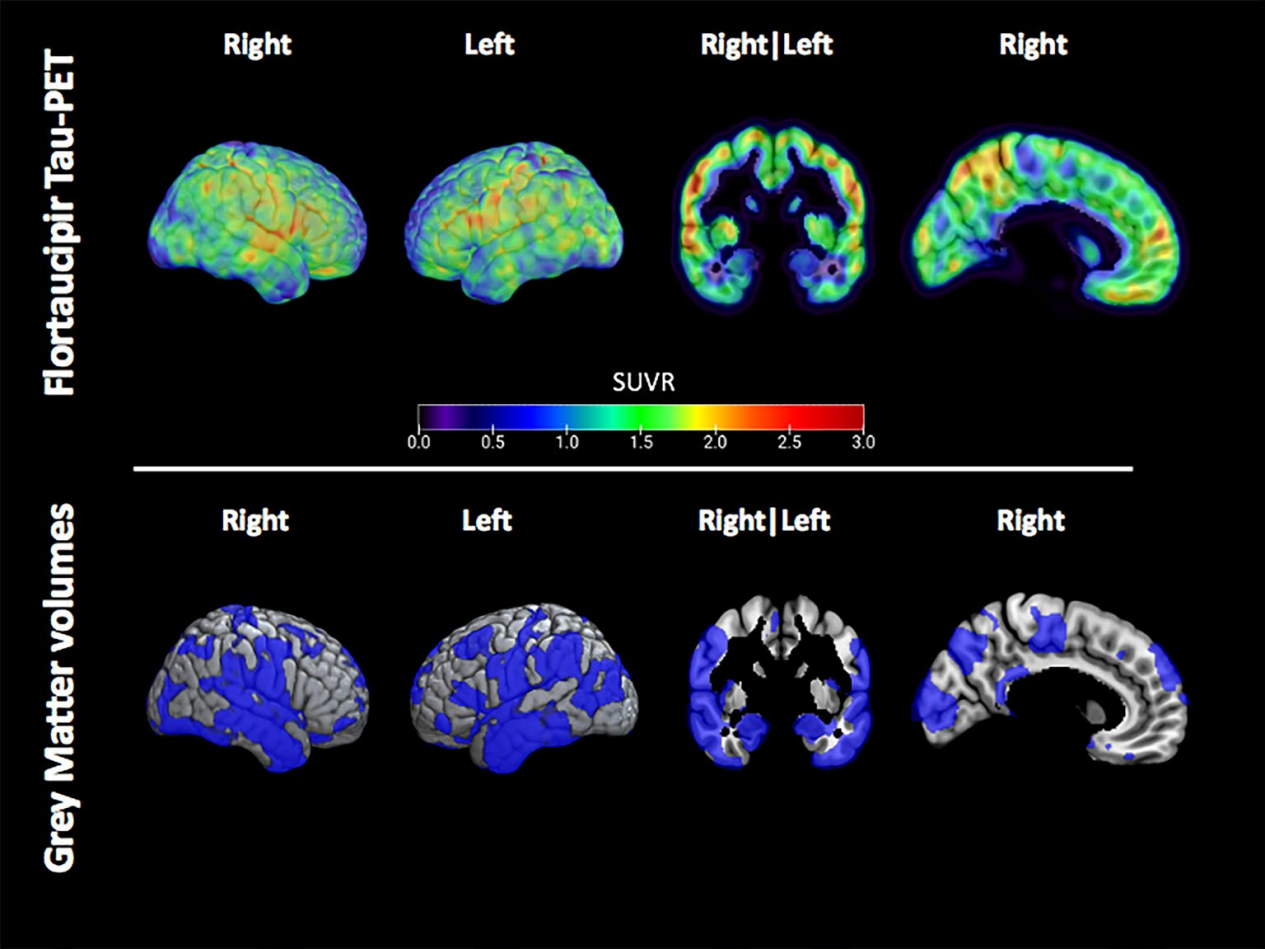
Example of the flortaucipir tau-PET signal and distribution of low grey matter volume in a single former athlete. Spatial maps of the flortaucipir tau-PET SUVR (top) and the reduced grey matter volumes showed in blue and defined as voxels with a w-score of > 1.5 below the mean (bottom) in a single former athlete

Age and intracranial volume adjusted grey matter w-score values were significantly lower in tau-PET positive voxels, compared to tau-PET negative voxels (− 0.37 ± 0.41 vs. − 0.31 ± 0.37, paired *p* = 0.006, BCa 95% CI of the mean difference 0.02–0.10). An example of spatial maps of flortaucipir tau-PET SUVR values and reduced volumes in the grey matter of a single former athlete is shown in Fig. 2.

### Tau-PET quartiles

**Fig. 3:**
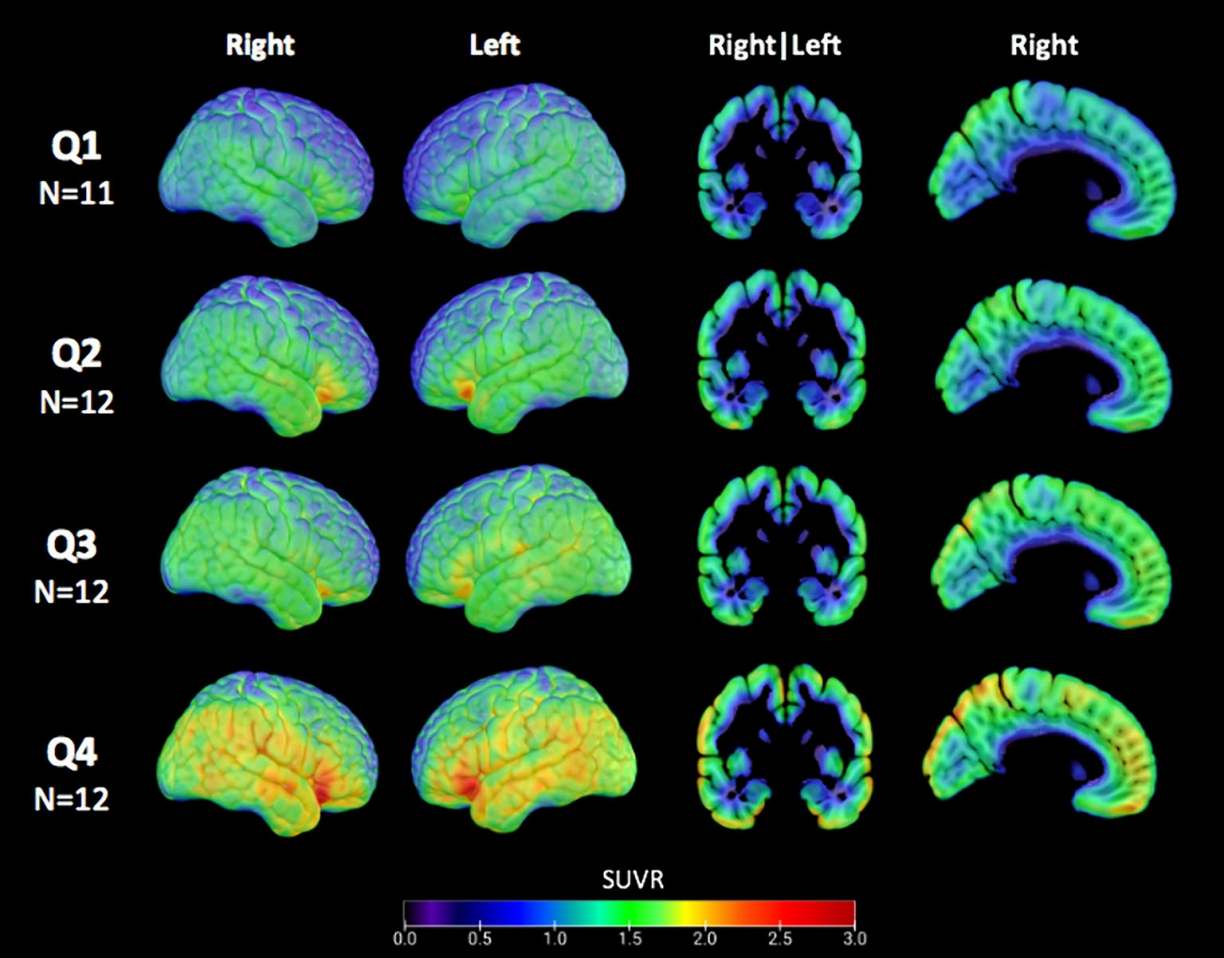
Mean tau-PET SUVR values across quartiles

The average tau-PET SUVR images from the first quartile (corresponding to the lowest % tau-PET positivity) through to the fourth quartile (corresponding to the highest % tau-PET positivity) are visualized in Fig. 3. The tau-PET showed a distinct spatial pattern extending from the lateral frontal and temporal lobes posteriorly, increasing in signal intensity across quartiles. Supplementary Table 2 shows grey matter areas with a significant increase in tau-PET % voxel positivity across the quartiles, controlled for age, PET scanner, scan duration, and for multiple comparisons using the Bonferroni method. All significant areas showed an increase in tau-PET % positivity of > 7%; however, hippocampus, putamen, and caudate showed no significant increases in tau-PET % positivity across quartiles.

### Tau-PET and neuropsychological assessments

Pearson partial correlations between tau-PET grey matter % positivity and tau-PET grey matter SUVR values and neuropsychological composite scores are included in Table 2, corrected for tau-PET scanner, scan duration, age, education, and adjusted for multiple comparisons using Bonferroni. Lower memory composite scores were significantly correlated with higher tau-PET grey matter % positivity (r =  − .363, *p* = .027, 95% BCa CI − .603 to − .116) and higher tau-PET grey matter SUVR values (r =  − .322, *p* = .05, 95% BCa CI − .561 to − .069). The memory score composite was more strongly associated with tau-PET grey matter % positivity than with the tau-PET SUVR values. There were no significant associations between tau-PET measures and attention and speed of processing or executive function composite scores.

**Table 2:** Pearson’s correlation between tau-PET positivity and neuropsychological assessments

	Tau-PET % Positive		Tau-PET SUVR	
	*r* (95% BCa CI)	Unadjusted *p*	*r* (95% BCa CI)	Unadjusted *p*
Memory composite score	− .363 (− .603 to − .116)	***.027***	− .322 (− .561 to − .069)	***.05***
*N*	46		46	
Speed of processing composite score	− .033 (− .368 to .346)	.8	− .005 (− .333 to .390)	1
*N*	46		46	
Executive functioning composite score	− .026 (− .415 to .356)	.9	− .010 (− .405 to .330)	1
*N*	45		45	

Partial correlation adjusted for age, education, tau-PET scanner, and tau-PET scan duration. 95% bias-corrected accelerated (BCa) bootstrapped CIs with 1000 repetitions were performed. *N* under the respective assessments represents the number of participants who completed the assessments

*P* values in *bold italics* are considered significant

*RAVLT* Rey Auditory Verbal Learning Test, *RVDLT* Rey Visual Design Learning Test, *SDMT* Symbol digit modalities test, *TMT* Trail making test, *WCST* Wisconsin card sorting test

## Discussion

This study investigated the relationship between flortaucipir tau-PET and grey matter volumes and neuropsychological functioning in a cohort of AD biomarker-negative retired athletes. We found that tau-PET positive regions had lower grey matter volumes than did the tau-PET negative regions, suggesting an underlying pathophysiological process causing grey matter atrophy. The spatial pattern of tau-PET signal involved mostly frontal and temporal regions and extended posteriorly with increasing tau-PET % positivity. This is consistent with postmortem studies that reported tau pathology of CTE predominantly in frontal and temporal regions while sparing the primary visual cortex [29, 30]. In our study we similarly saw relative sparing of the tau-PET signal in the primary visual cortex; however, while the current CTE staging emphasizes involvement of the dorsolateral prefrontal cortex[31], our data showed increased tau-PET signal specifically in the inferior frontal gyrus and orbitofrontal cortex.

Previous literature on flortaucipir tau-PET in athletes reports increased signal in frontotemporal regions in a patchy pattern; however, specific regions mentioned differ between studies. In amyloid-negative National Football League (NFL) players, one single-participant case study reported an increased tau-PET signal in bilateral cingulate, occipital, orbitofrontal, and temporal cortices [4], while another noted subcortical tau-PET signal localized to basal ganglia and substantia nigra [5]. A larger investigation in 26 AD biomarker-negative former NFL players reported higher levels of tau-PET in bilateral superior frontal, bilateral medial temporal, and left parietal areas, compared to controls [6]. The results of our study are similar to a recent paper comparing near end-of-life flortaucipir tau-PET to postmortem CTE tau pathology in six former NFL players where a strong association between tau-PET ligand binding and p-tau density on neuropathology in cortical and limbic areas was reported. Similarly to our study, they found the highest tau-PET signal in orbitofrontal and superior temporal regions that closely followed neuropathological findings, with tau-PET ligand binding pattern in the hippocampus being inconsistent with neuropathology (i.e., some participants including controls had increased tau-PET in hippocampus and some did not, irrespective of whether there was presence or absence of tau pathology in hippocampus) [3]. We also did not find a consistent increase in tau-PET signal across the quartiles in the hippocampus (there was also no significant increase in tau-PET signal across the quartiles in basal ganglia) which could be due to these regions being known off-target binding sites of the flortaucipir tau-PET tracer [32, 33].

Our study did not find any associations between the number of concussions, years of total or professional play, and tau-PET measures, which is consistent with another study in the current literature [34] although some studies have found associations between tau pathology or tau-PET signal and years of play [6, 35]. Other studies found that playing football after high school corresponds to an increased risk of developing CTE [29, 36]. While other studies included athletes across all levels of play, our cohort consisted mainly of former professional players. Lack of athletes from different levels of play in our cohort could have diluted any association between tau-PET measures and years of play in our study. Additionally, subconcussive hits are also believed to play a role in CTE pathology [37], but are difficult to quantify.

Even though our study excluded anyone with known neurological or neurodegenerative conditions and even anyone with AD biomarkers and normal function, we found that increased tau-PET signal in our cohort was significantly associated with decreased memory performance. Concussions have been shown to alter functional networks involving visual attention and working memory [38], and retired players with histories of concussions were found to have worse performance on learning tasks [39, 40]. These deficits in learning and memory have been associated with functional and structural changes in multiple areas including the hippocampi and orbitofrontal cortex [38, 39]. Our memory tasks included visual and verbal tests and the association between high tau-PET and decreased memory was driven predominantly by the learning scores of our memory assessments. The findings of increased tau-PET signal in the orbitofrontal cortex of our retired players could be an important indicator that tau deposition in those areas leads to reduced memory functioning [41]. The orbitofrontal cortex is also an area affected in mild TBIs and is implicated in behavioral and neuropsychiatric changes, including those seen in CTE [42].

The meaning of flortaucipir binding in former contact sports athletes and its ability to reflect CTE has been a matter of debate. While two studies with a total of seven cases comparing premortem flortaucipir tau-PET signal with postmortem tau pathology of CTE reported a moderate-strong association between tau-PET tracer binding and tau pathology [3, 8], an autoradiography study of five CTE cases comparing postmortem flortaucipir tracer binding with tau immunostaining found no association [7]. Thus, there may be differences in tracer binding in live tissues when compared to postmortem binding, or that higher sample sizes are needed to further compare premortem flortaucipir tau-PET to neuropathology.

Even though our study controlled for the presence of AD pathology, we do not have neuropathology available and do not know who or if any of the retired athletes in our study have CTE pathology, although many of them have indicated their desire to donate their brains to the Canadian Concussion Centre Brain Bank. However, our findings of decreased grey matter volumes in regions with high tau-PET signal are suggestive of an underlying pathological process, and since these patients are AD biomarker negative and have no clinical or imaging features of other tauopathies, one needs to consider CTE a possibility. Concussions and subconcussive blows have been shown to be associated with metabolic and structural changes specifically in frontal and temporal areas [43, 44]. These regions are highly vulnerable to injury as a result of head impacts as they are situated in the anterior and cranial fossa of the skull. This positioning of the frontal and temporal lobes creates surface areas of contact between the brain and skull where mechanical deformation injures the brain [45].

The pathognomonic CTE lesion has been defined as “an accumulation of abnormal hyperphosphorylated tau in neurons and astroglia distributed around small blood vessels at the depth of cortical sulci and in an irregular pattern.”[46] In the early CTE stages (I–II), the neurofibrillary tangles of tau are focal and are usually in the frontal cortex. In the later CTE stages (III–IV), the tangles are widespread throughout cortical and subcortical areas [47]. The patchy and focal tau inclusions during the early CTE stages could be the reason why previous studies only found a moderate relationship between flortaucipir tau-PET and CTE pathology in later stages. As tau is found at the depth of cortical sulci, it poses challenges to capturing this pathology using tau-PET when only a few inclusions are present and could be easier to see with tau-PET imaging when the tau tangles are more widespread as in later CTE stages. Also, it should be noted that flortaucipir has limited specificity to 4R tau isoforms in neurodegenerative diseases. While CTE is a mixed 3R/4R tauopathy, there is evidence to suggest that mixed tauopathies like CTE begin as a 4R predominant pathology which evolves into 3R predominant in the later stages of the disease [48]. This change in tau isoforms in CTE could be the reason behind flortaucipir’s increasing specificity to tau pathology in later CTE stages.

There are several limitations to the current study, beginning with the absence of brain pathology which does not allow us to draw any conclusions about the definitive source of the tau-PET tracer binding. There is also a potential for recall bias in the self-report questionnaires for concussion history. The small number of females in the athletes cohort limits the generalizability of our results, and the unequal number of males and females between athletes and controls could have affected our findings on grey matter volumes. While most of the biofluid analysis for the presence of AD pathology has been completed at the same time or after tau-PET, some of the retired athletes that completed their biofluid visit prior to tau-PET. This could potentially limit the ability to identify the presence of AD pathology among our participants although it is well known that CSF biomarkers of neurodegeneration are positive prior to PET biomarkers and many years before clinical symptoms appear [49, 50]. Finally, our cohort of retired athletes is highly heterogeneous with respect to ages, sports history, and tau-PET acquisition, which could have confounded our findings.

In conclusion, Flortaucipir tau-PET is able to identify neuropathological changes in retired athletes at risk of neurodegeneration, as seen by its relationship with grey matter volumes and memory functioning. However, more studies examining the relationship between flortaucipir tau-PET and postmortem CTE pathology are needed to identify its specificity to CTE pathology.

## Supplementary Information

Below is the link to the electronic supplementary material.

Supplementary file1 (DOCX 19 kb)

## Data Availability

Anonymized data not published within this article will be made available by request from any qualified investigator.
