# Supplementary material for: 18F-Flortaucipir (AV1451) imaging identifies grey matter atrophy in retired athletes
Source: J Neurol. 2024 Jul 22;271(9):6068–79. doi: 10.1007/s00415-024-12573-0 (PMC11377597; doi:10.1007/s00415-024-12573-0)
Supplement: Supplementary file 1 — Supplementary file1 (DOCX 19 kb) [file 415_2024_12573_MOESM1_ESM.docx]

**Supplementary Table 1.** Participants from the retired athletes' cohort that had biofluid analysis completed during >1 visit.

| ID | CSF AD Markers | Plasma p-tau181  Time 1 | Plasma p-tau181  Time 2 |
| --- | --- | --- | --- |
| Participant 1 | -ve | -ve |  |
| *Visit* | *bl* | *bl* |  |
| Participant 2 | -ve | -ve |  |
| *Visit* | *bl* | *bl* |  |
| Participant 3 | -ve | -ve |  |
| *Visit* | *bl* | *bl* |  |
| Participant 4 | -ve | -ve | +ve |
| *Visit* | *bl* | *4 yrs pre-bl* | *4 yrs f/u* |
| Participant 5 | -ve | -ve | -ve |
| *Visit* | *bl* | *2 yrs pre-bl* | *2 yrs f/u* |
| Participant 6 |  | -ve | -ve |
| *Visit* |  | *2 yrs pre-bl* | *bl* |
| Participant 7 | -ve | -ve |  |
| *Visit* | *bl* | *2 yrs pre-bl* |  |
| Participant 8 | -ve | -ve |  |
| *Visit* | *bl* | *2 yrs pre-bl* |  |
| Participant 9 | -ve | -ve |  |
| *Visit* | *2 yrs f/u* | *2 yrs pre-bl* |  |
| Participant 10 |  | -ve | -ve |
| *Visit* |  | *2 yrs pre-bl* | *bl* |
| Participant 11 |  | -ve | -ve |
| *Visit* |  | *6 yrs pre-bl* | *2 yrs f/u* |

Results of the biofluid (blood and CSF) results for the retired athletes that had completed more than one biofluid visit. “+ve” refers to the biomarker being positive for the presence of AD pathology. “-ve” refers to the biomarker being negative for the presence of AD pathology. CSF AD markers included total tau, phosphorylated (p-tau) 181, and amyloid beta 42. Visit refers to the time of the biofluid collection visit compared to the tau-PET visit: 1) “N” yrs pre-bl – the biofluid visit was completed “N” years prior to tau-PET; 2) bl – the biofluid visit was completed at the same baseline visit as tau-PET; 3) “N” yrs f/u – the biofluid visit was completed “N” years after the tau-PET visit. CSF, cerebrospinal fluid; AD, Alzheimer’s disease.

**Supplemental Table 2.** Regions of interest ordered by the highest level of tau-PET % voxel positivity.

| **Region** | **BCa Unstandardized Coefficient** | **BCa 95% CI Lower** | **BCa 95% CI Higher** | **Model *p*** |
| --- | --- | --- | --- | --- |
| precuneus | 13.427 | 11.082 | 15.827 | ***<0.001*** |
| angular gyrus | 13.023 | 10.408 | 15.895 | ***<0.001*** |
| middle occipital gyrus | 12.855 | 10.027 | 15.537 | ***<0.001*** |
| supramarginal gyrus | 12.423 | 9.744 | 15.088 | ***<0.001*** |
| cuneus | 12.279 | 9.109 | 15.523 | ***<0.001*** |
| superior temporal gyrus | 12.262 | 10.303 | 14.34 | ***<0.001*** |
| inferior frontal gyrus | 12.141 | 9.736 | 14.514 | ***<0.001*** |
| middle frontal gyrus | 12.13 | 9.851 | 14.295 | ***<0.001*** |
| middle temporal gyrus | 12.059 | 9.625 | 14.484 | ***<0.001*** |
| superior frontal gyrus | 11.622 | 9.653 | 13.453 | ***<0.001*** |
| inferior temporal gyrus | 10.864 | 7.441 | 14.34 | ***<0.001*** |
| superior occipital gyrus | 10.818 | 7.175 | 14.491 | ***<0.001*** |
| insular cortex | 10.72 | 8.091 | 13.208 | ***<0.001*** |
| lateral orbitofrontal gyrus | 10.716 | 8.243 | 13.305 | ***<0.001*** |
| middle orbitofrontal gyrus | 10.641 | 7.715 | 13.246 | ***<0.001*** |
| precentral gyrus | 10.629 | 9.13 | 12.049 | ***<0.001*** |
| superior parietal gyrus | 10.626 | 8.122 | 13.237 | ***<0.001*** |
| postcentral gyrus | 10.112 | 7.867 | 12.314 | ***<0.001*** |
| cingulate gyrus | 9.942 | 7.914 | 12.362 | ***<0.001*** |
| inferior occipital gyrus | 9.583 | 6.726 | 12.503 | ***<0.001*** |
| lingual gyrus | 8.386 | 6.007 | 11.174 | ***<0.001*** |
| parahippocampal gyrus | 7.461 | 4.631 | 10.444 | ***<0.001*** |
| fusiform gyrus | 7.274 | 4.881 | 9.808 | ***<0.001*** |
| hippocampus | 4.542 | 2.037 | 7.745 | 0.007 |
| putamen | 4.001 | 0.682 | 6.958 | 0.024 |
| caudate | 1.984 | 0.448 | 3.344 | 0.014 |

Multiple linear regressions with region-specific voxel extent as a dependent variable and quartile group and age as independent variables. The regions were then ordered based on unstandardized coefficients. To control for influence of outliers, linear regressions were performed with 95% bias-corrected accelerated (BCa) bootstrapped CIs with 2000 repetitions stratified by quartile group. Results were controlled for multiple comparisons using Bonferroni method. *P* values of <.0019 were considered significant after controlling for multiple comparisons, and are shown in ***bold italics***.
